# One-Pot Facile Synthesis of Noble Metal Nanoparticles Supported on rGO with Enhanced Catalytic Performance for 4-Nitrophenol Reduction

**DOI:** 10.3390/molecules26237261

**Published:** 2021-11-30

**Authors:** Xiaolong Zhang, Shilei Jin, Yuhan Zhang, Liyuan Wang, Yang Liu, Qian Duan

**Affiliations:** 1 School of Materials Science and Engineering, Changchun University of Science and Technology, Changchun 130022, China; zhangxiaolong@jlnu.edu.cn; 2 Key Laboratory of Functional Materials Physics and Chemistry of the Ministry of Education, Jilin Normal University, Changchun 130103, China; jinshilei2001@163.com (S.J.); zhangyuhan193@163.com (Y.Z.); wly18629899813@163.com (L.W.); liuyang@jlnu.edu.cn (Y.L.)

**Keywords:** 4-nitrophenol, NaBH_4_, noble metal nanoparticles, catalytic reduction

## Abstract

In this study, reduced graphene oxide (rGO)-supported noble metal (gold, silver, and platinum) nanoparticle catalysts were prepared via the one-pot facile co-reduction technique. Various measurement techniques were used to investigate the structures and properties of the catalysts. The relative intensity ratios of *I_D_/I_G_* in rGO/Au, rGO/Ag, rGO/Pt, and GO were 1.106, 1.078, 1.047, and 0.863, respectively. The results showed the formation of rGO and that noble metal nanoparticles were decorated on rGO. Furthermore, the catalytic activities of the designed nanocomposites were investigated via 4-nitrophenol. The catalysts were used in 4-nitrophenol reduction. The catalytic performance of the catalysts was evaluated using the apparent rate constant k values. The k value of rGO/Au was 0.618 min^−1^, which was higher than those of rGO/Ag (0.55 min^−1^) and rGO/Pt (0.038 min^−1^). The result proved that the rGO/Au catalyst exhibited a higher catalytic performance than the rGO/Ag catalyst and the rGO/Pt catalyst. The results provide a facile method for the synthesis of rGO-supported nanomaterials in catalysis.

## 1. Introduction

Nanoparticles (NPs) of noble metals (gold, silver, and platinum) have attracted greater interest from researchers than bulk metals because of their unique properties, such as electronic, optical, magnetic, and catalytic properties [1,2,3]. Therefore, noble metal NPs have been widely used in many applications, such as catalysis, optical, magnetic, biomedical, Raman, and other fields [4,5,6]. In recent years, organic pollutants from industrial waste have seriously affected the environment and human health; thus, the removal of industrial waste has received great attention [7,8]. As a high-priority pollutant, 4-nitrophenol (4-NP) is widely distributed in the environment; it is the most refractory pollutant negatively affecting the health of humans and animals [9,10,11,12]. Thus, it is important to remove or reduce the presence of 4-NP in the environment. The reduction product of 4-NP is 4-aminophenol (4-AP); 4-AP has been extensively applied in medicine, dyes, and many other fields [13,14,15]. Various methods have been developed to remove or reduce 4-NP, such as adsorption, photodegradation, solid-phase extraction, electrolytic reduction, and biodegradation [16,17,18]. However, these methods have some problems, such as long duration, high operational cost, high energy consumption, etc. [19,20,21]. The catalytic reduction method (4-NP reduction by catalysts with sodium borohydride) has received significant attention due to its low cost, high conversion efficiency, low energy consumption, etc. [21,22,23].

Noble metal NPs (gold [24], silver [25], palladium [26], and platinum [27]) have been synthesized using various methods for 4-NP reduction, and they exhibit excellent catalytic activity. However, their low abundance and aggregation limit their application in various fields [28]. As carbon-based materials, graphene and reduced graphene oxide (rGO) have attracted great attention from researchers due to their chemical stability, excellent adsorption performance, etc. [29,30,31,32]. Therefore, graphene and rGO have been used as supports for catalysts to enhance performance of the catalytic reduction of organic pollutants. From the perspective of catalysis, the carbon materials supporting the noble metal catalysts can effectively enhance the catalytic performance and remove the original pollutions from the environment in practical applications, which may be due to their unique structural characteristics and the synergistic effects. In this work, rGO/Au, rGO/Pt, and rGO/Ag were prepared via the controllable one-step route. The enhanced catalytic performances of the composite materials were investigated by 4-NP reduction. This work promotes the application of the reduced graphene oxide-based nanocomposite catalysts in the catalytic field.

## 2. Results and Discussion

### 2.1. Characterization and Properties of the rGO/Au, rGO/Ag, and rGO/Pt Composites

**Figure 1:**
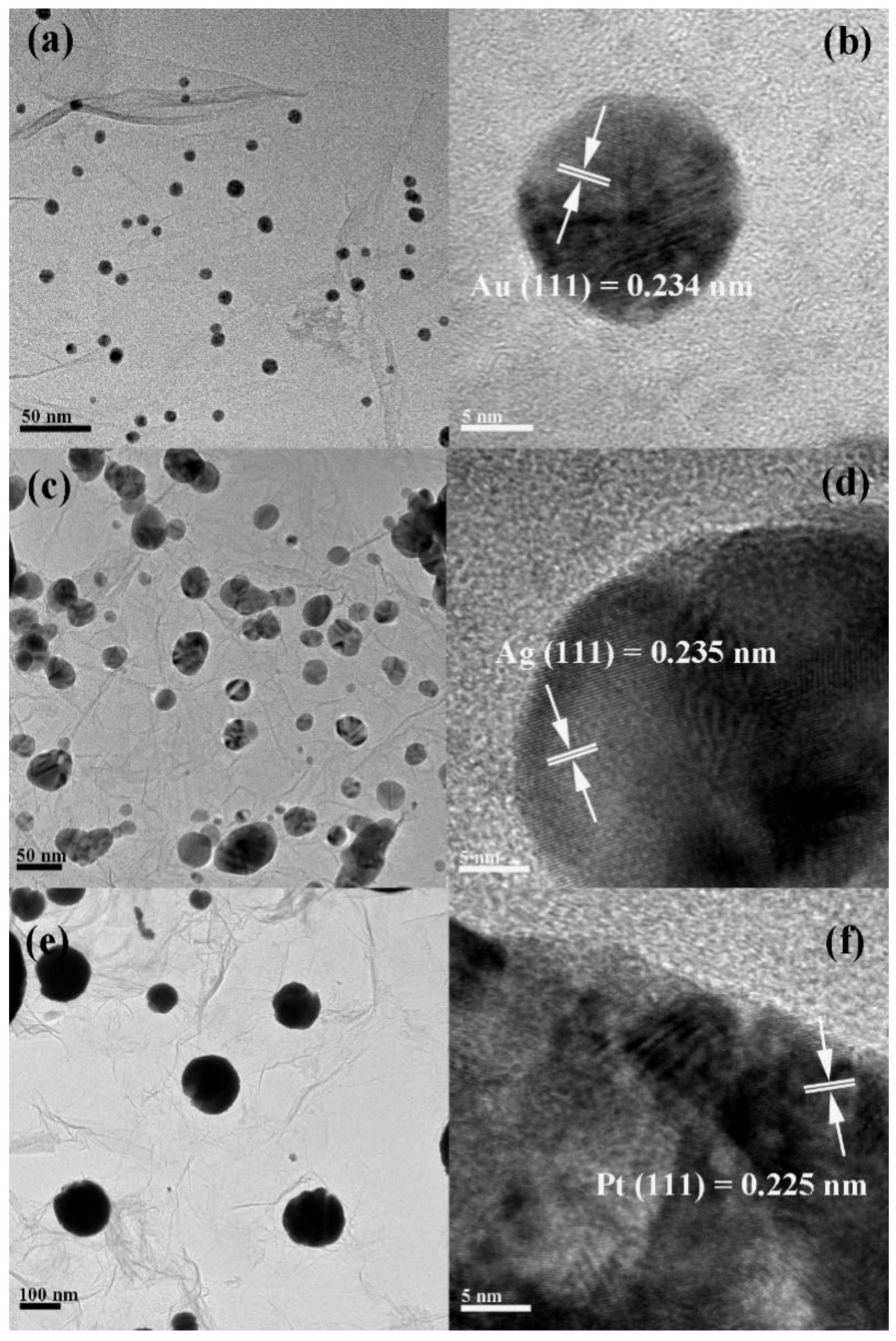
(**a**,**c**,**e**) TEM images and (**b**,**d**,**f**) HRTEM images of rGO/Au, rGO/Ag, and rGO/Pt.

**Figure 2:**
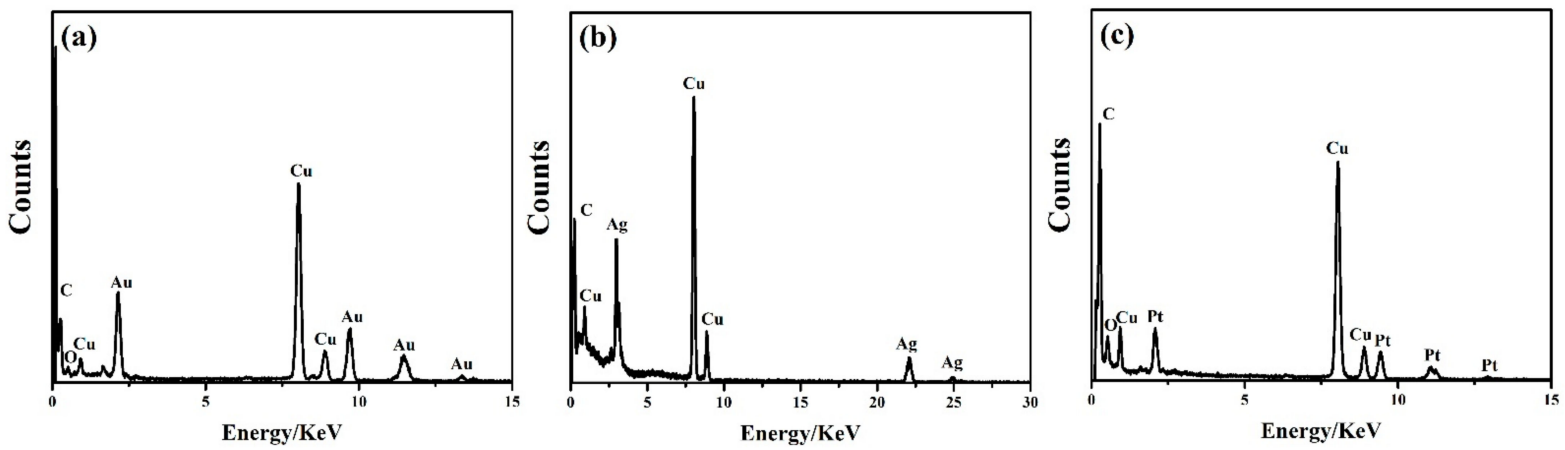
EDX spectra of (**a**) rGO/Au, (**b**) rGO/Ag, and (**c**) rGO/Pt.

TEM, HRTEM, and EDX were used to investigate the morphology and structure of all the samples. Figure 1 and Figure 2 show the TEM images, the HRTEM images, and the EDX spectra of the composites. For all the composites, the NPs are nearly spherical, and the rGO is decorated with noble metal NPs with different crystalline size distributions (Figure 1). These results confirm the formation of composites. Moreover, Figure 1 shows that the crystalline size of gold NPs is smaller than that of silver NPs and platinum NPs. The small size of gold NPs on the rGO surface may have contributed to the enhancement of the catalytic performance in the catalytic reduction reaction. According to the HRTEM results (Figure 1), the lattice spacings of the noble metal NPs are 0.234, 0.235, and 0.225 nm, which are related to the lattice plane (111) of gold [33], silver [34], and platinum, respectively [35]. The corresponding EDX spectra of the composites (Figure 2a–c) also confirm the formation of the composites and the existence of carbon, copper, oxygen, gold, silver, and platinum.

**Figure 3:**
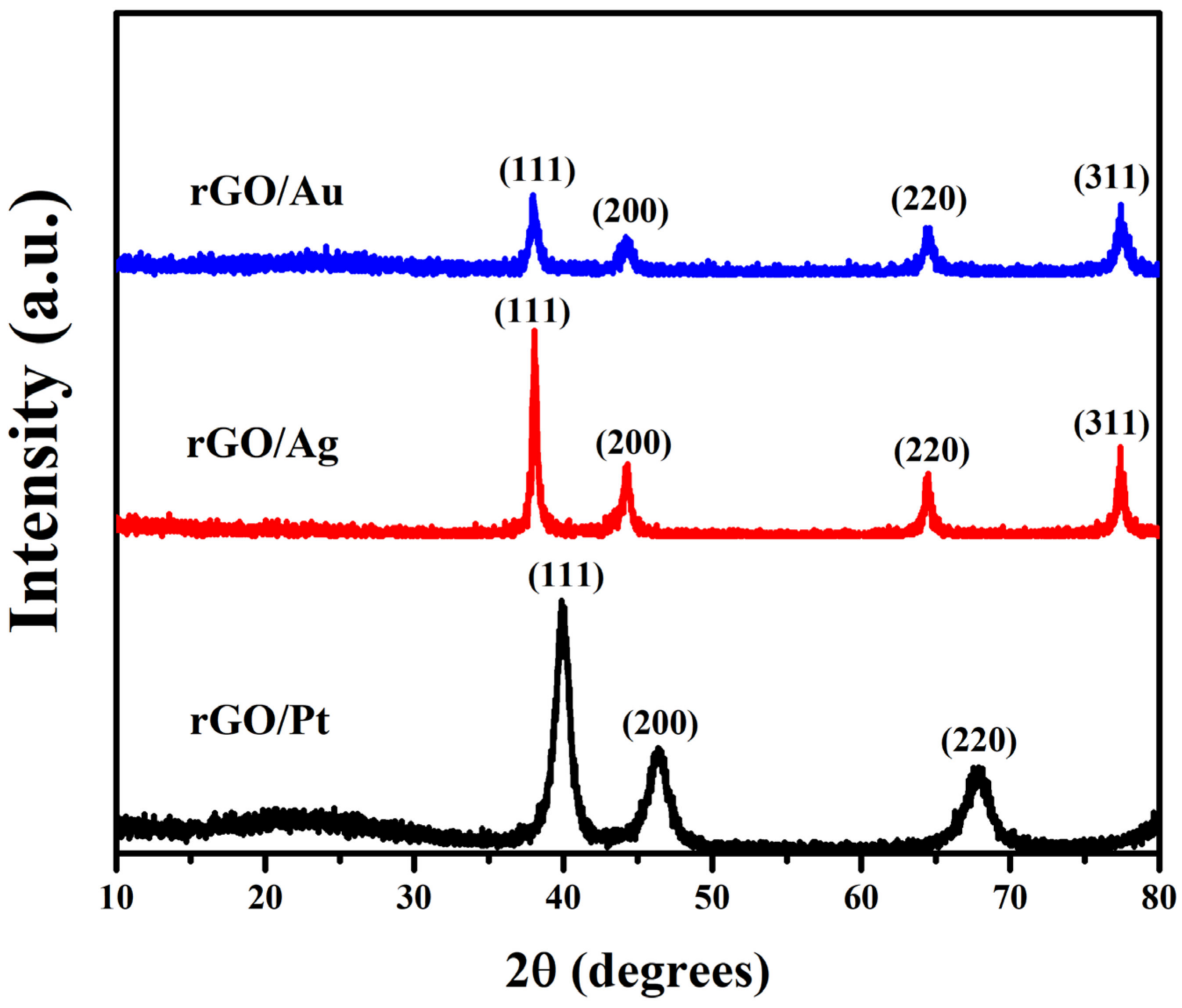
XRD patterns of the composites.

The structures of the composites were investigated via XRD as shown in Figure S1 and Figure 3. Figure S1 displays the XRD patterns of GO and rGO. Figure 3 shows that the diffraction peaks of the rGO/Au composite at 38.12°, 44.33°, 64.53°, and 77.51° correspond to the planes of face-centered cubic (FCC) gold nanoparticles (NPs) [36,37]. The XRD patterns of the rGO/Ag and rGO/Pt composites display four and three diffraction peaks, respectively, which confirm the formation of FCC silver NPs and FCC platinum NPs [38,39]. Furthermore, the intensities of the XRD diffraction peaks for the composites follow the sequence rGO/Au < rGO/Ag < rGO/Pt, which confirms that the crystalline size of Au is smaller than that of Ag and Pt. The XRD results are in accordance with the results of TEM. Furthermore, no obvious diffraction peaks of rGO were detected in the XRD patterns of the rGO/Au and rGO/Ag composites, which may be due to the lower content of rGO or the weaker diffraction peak intensity of rGO when compared to the diffraction peaks of the noble metal NPs. In addition, there was no obvious change in the crystalline structures of the noble metal NPs, which confirms that rGO may only provide a platform for the growth of noble metal NPs [40].

**Figure 4:**
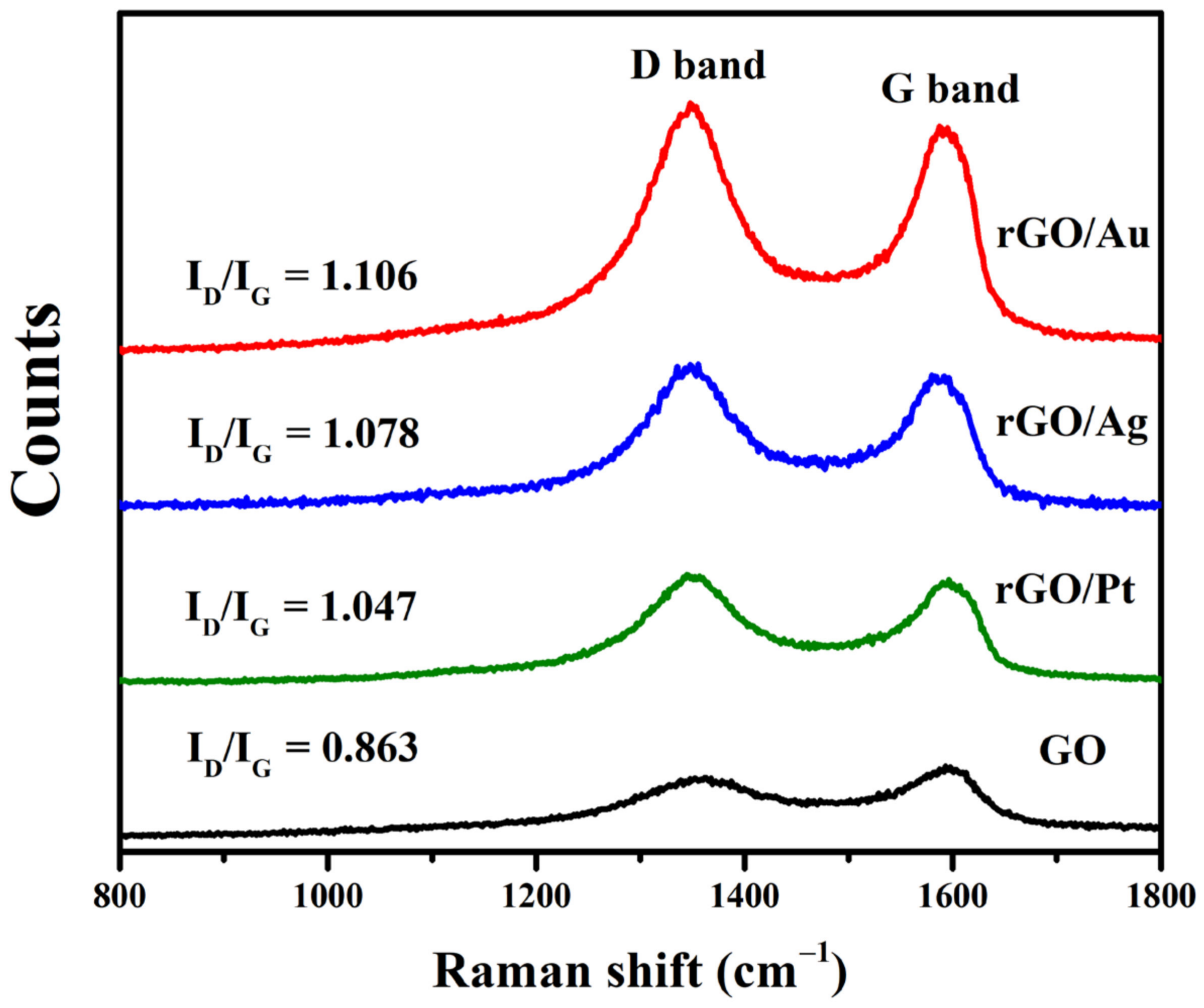
Raman spectra of the composites and GO.

Raman spectra were applied to further investigate the effective reduction of GO into rGO. Figure 4 displays the Raman spectra of all the samples, showing that two strong peaks were detected in the range of 1300–1600 cm^−1^ for all the samples. The peaks are assigned to the characteristic peaks of carbon materials (D band and G band). It is well-known that the relative intensity ratio of *I_D_/I_G_* can represent the defect density of samples. The relative intensity ratios of *I_D_/I_G_* in rGO/Au, rGO/Ag, rGO/Pt, and GO were 1.106, 1.078, 1.047, and 0.863, respectively. The values of *I_D_/I_G_* for all the samples are higher than that of GO, which confirms the successful reduction of GO via a facile method [41].

**Figure 5:**
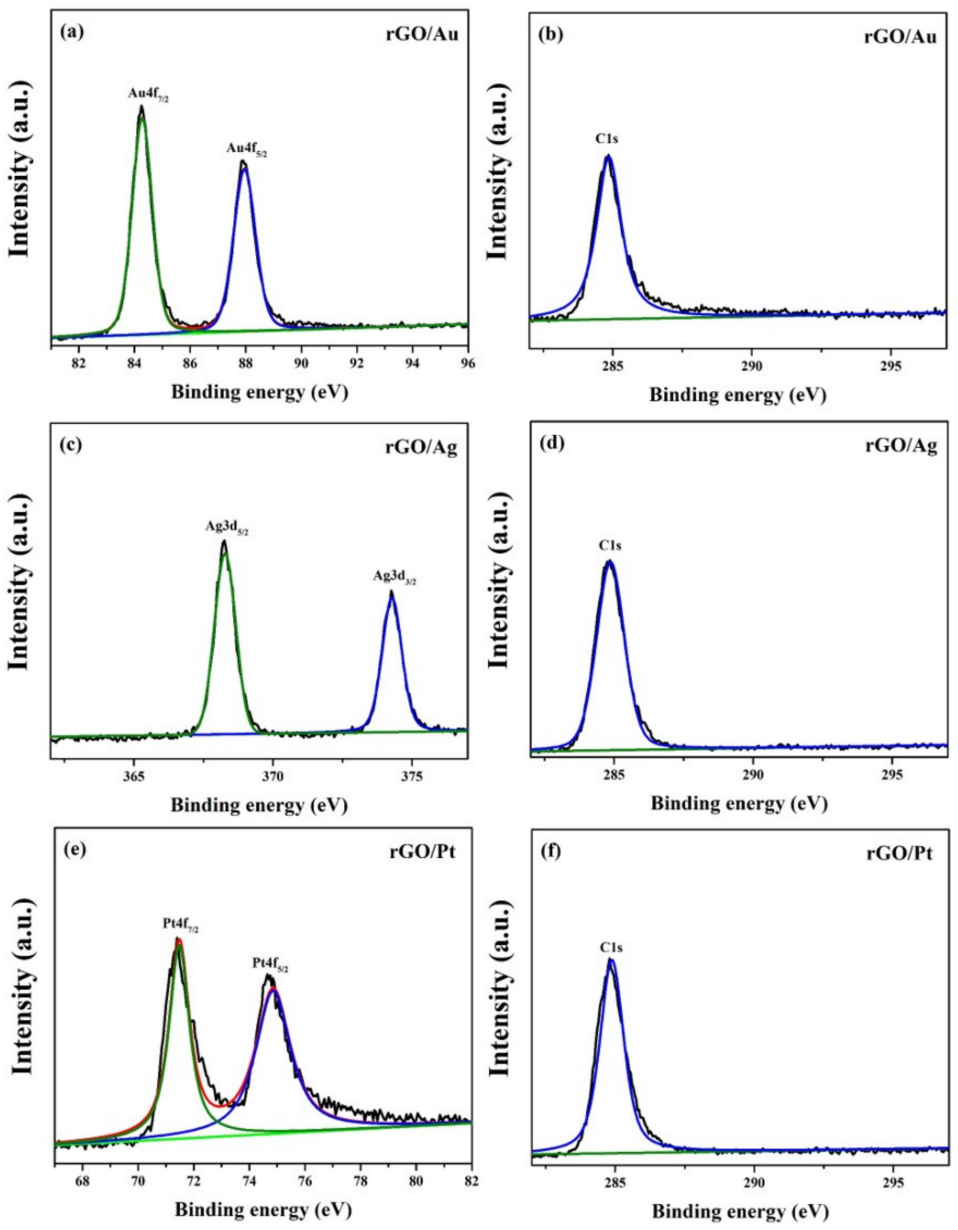
XPS of (**a**) Au4f, (**c**) Ag3d, (**e**) Pt4f, and (**b**,**d**,**f**) C1s of the composites.

The chemical composition and valence state of all the samples were investigated by means of XPS (Figure 4 and Figure S2). Figure S2 confirms the existence of the Au, Ag, Pt, C, and O elements in the composites, which is in agreement with the EDX results. For the spectrum of Au4f in Figure 5a, the peaks located at about 84.2 and 87.9 eV are assigned to Au4f_7/2_ and Au4f_5/2_, respectively [42]. As shown in Figure 5c, there are two peaks in the high-resolution spectrum of Ag3d which correlate with Ag3d_5/2_ and Ag3d_3/2_ [43]. Meanwhile, the peaks of Pt4f, as shown in Figure 5e, constitute Pt4f_7/2_ and Pt4f_5/2_, which are located at about 71.3 and 74.6 eV, respectively [44]. According to the above results, the peaks of Au4f, Ag3d, and Pt4f are in accordance with the characteristic peaks for metallic Au^0^, Ag^0^ and Pt^0^, which confirm the formation of gold NPs, silver NPs, and platinum NPs. The C1s spectra of all the samples are shown in Figure 5b,d,f. Only one peak was detected (located at about 284.8 eV), which corresponds to the functional group of carbon atoms (C–C). This confirms that GO had been reduced to rGO [39]. The XPS results are in accordance with the Raman results.

### 2.2. Catalytic Applications of the rGO/Au, rGO/Ag, and rGO/Pt Catalysts

**Figure 6:**
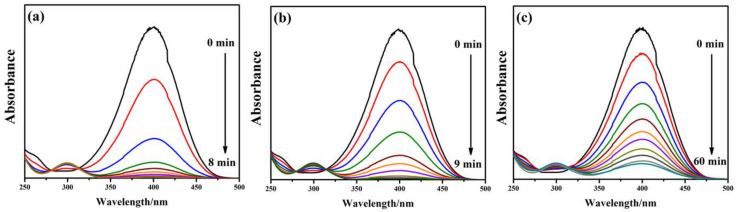
UV–vis absorption spectra of 4-nitrophenol reduction by (**a**) rGO/Au, (**b**) rGO/Ag, and (**c**) rGO/Pt.

**Figure 7:**
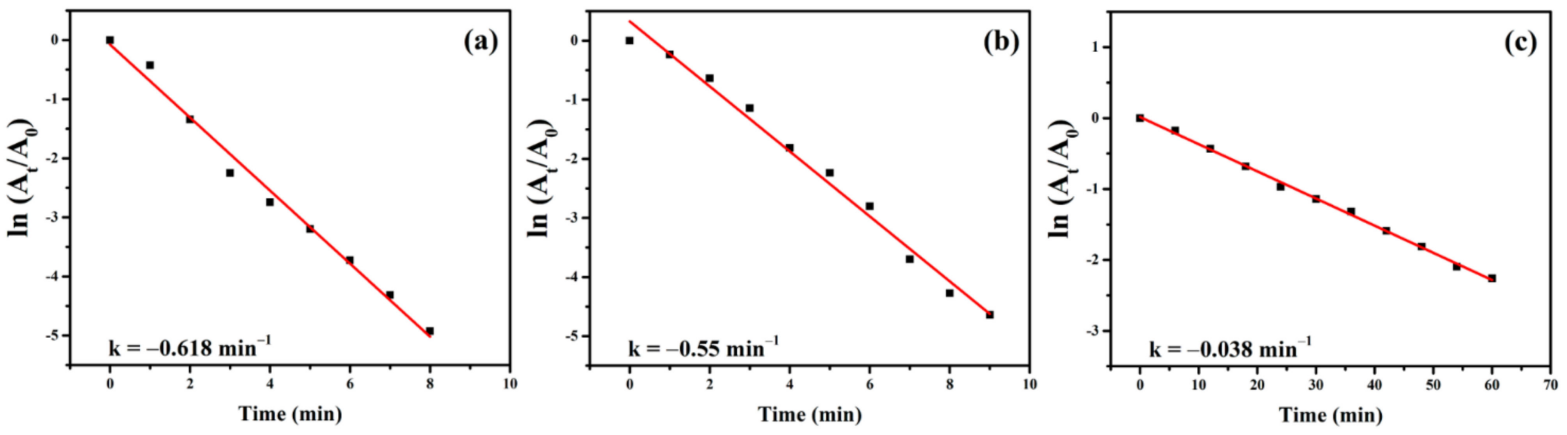
The plots of ln(A_t_/A_0_) versus reaction time for 4-nitrophenol reduction by (**a**) rGO/Au, (**b**) rGO/Ag, and (**c**) rGO/Pt.

**Figure 8:**
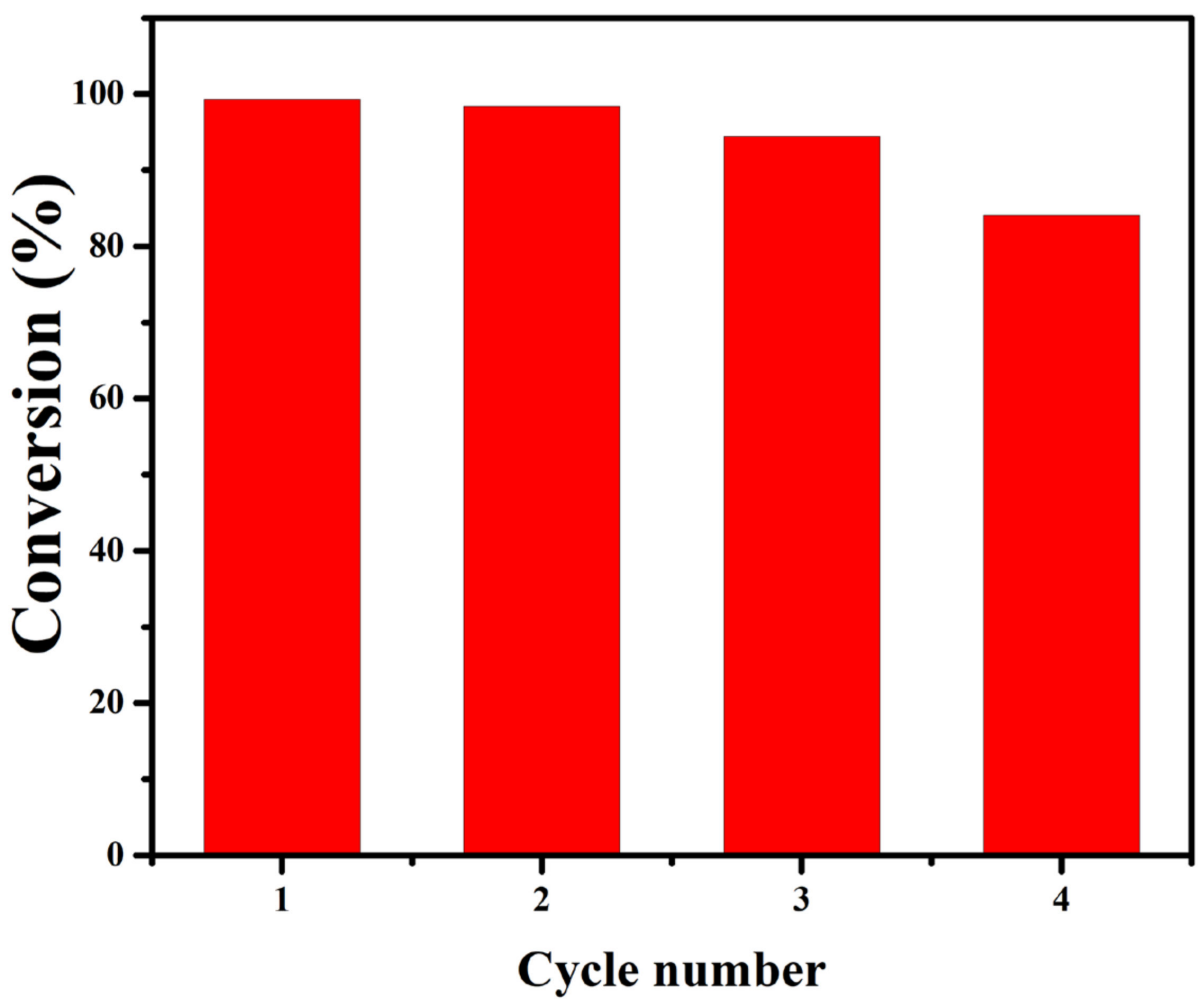
Reusability of the rGO/Au catalyst for 4-nitrophenol reduction.

As a model reaction, 4-NP reduction was employed to investigate the catalytic activity of all the samples. The reduction reaction process was monitored using a UV–visible spectrometer. The characteristic peak of original 4-NP is located at 317 nm. After a large amount of sodium borohydride (0.2 mmol) was added, the peak shifted to 400 nm and the color turned bright yellow. These phenomena occur because of the formation of 4-NP anions [45]. The change in the peak intensity was negligible if only NaBH_4_ was added. It confirms that the reaction does not proceed without the catalysts. However, after the addition of the catalysts, the intensities of the absorption peaks decreased significantly, and the peak of 4-AP appeared at 300 nm. Figure 6a–c shows the UV–vis spectra of the reaction by catalysts. Figure 6a shows that the reaction could be completed in 8 min with the rGO/Au catalyst, which is shorter than the completion time with the rGO/Ag (9 min) catalyst and the rGO/Pt (60 min) catalyst. Overall, the rGO/Au catalyst exhibited the best catalytic activity for 4-NP reduction. Furthermore, the catalytic performance of the catalysts was also evaluated by the values of the apparent rate constant k. During the reaction process, the concentration of NaBH_4_ was significantly higher than that of 4-NP; thus, it is reasonable to consider it to be a pseudo-first-order catalytic reduction. The apparent rate constant k was obtained according to the following equation: −ln(C_t_/C_0_) = −ln(A_t_/A_0_) = kt [46], where C_0_ and C_t_ are the concentrations of 4-NP at the beginning and at time t. A_0_ and A_t_ are the initial absorbance and the absorbance at time t, respectively. The linear correlations of ln(A_t_/A_0_) for all the catalysts versus time t are shown in Figure 7. The value of k was calculated by assuming a linear correlation between ln(A_t_/A_0_) versus time t. Figure 7 shows that the k values of the rGO/Au, rGO/Ag, and rGO/Pt catalysts were 0.618, 0.55, and 0.038 min^−1^, respectively. The value of k for the rGO/Au catalyst was larger than those of the rGO/Ag catalyst and the rGO/Pt catalyst, which confirms that the rGO/Au catalyst exhibited excellent catalytic performance. The superb catalytic activity of rGO/Au may be attributed to the following aspects: (1) the smaller crystalline size of gold NPs compared to silver NPs and platinum NPs and (2) the unique structural advantage provided by rGO [47]. A comparison of the catalytic performance of different catalysts is displayed in Table 1 [48,49,50,51]. Furthermore, recyclability of catalysts is one of the most important parameters for their application. Figure 8 shows the reusability of the rGO/Au catalyst for 4-NP reduction with NaBH_4_. The result shows that the conversion of 4-NP was still higher than 80% after four cycles, which proves the stability of the rGO/Au catalyst.

### 2.3. Possible Mechanism of 4-NP Reduction

**Figure 9:**
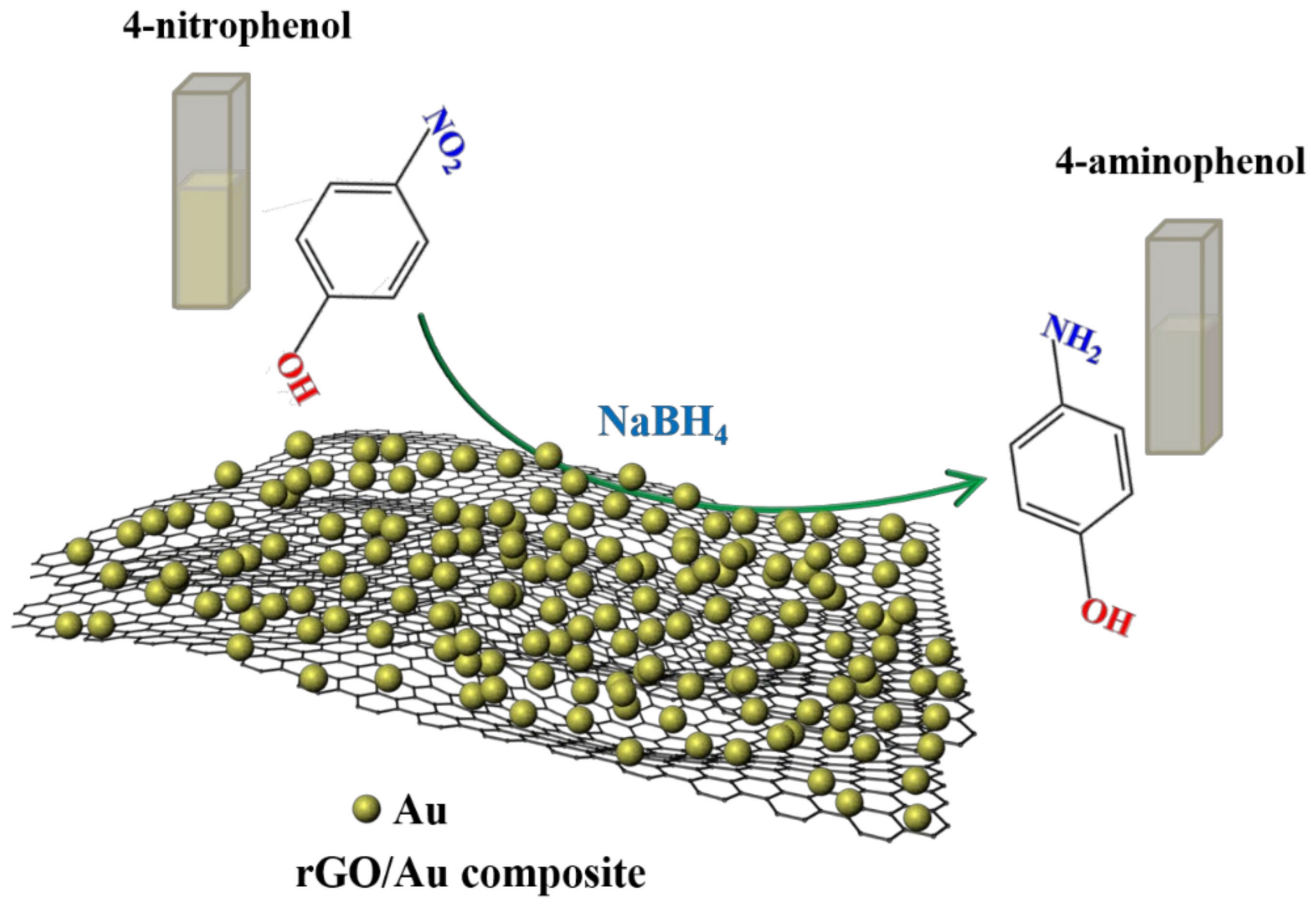
Possible catalytic mechanism for 4-nitrophenol reduction via the rGO/Au composite with NaBH_4_.

Figure 9 shows the possible mechanism of enhanced catalytic performance for 4-nitrophenol reduction. Generally, catalytic performance of catalysts may be attributed to two factors: (1) the adsorption capacity of the catalysts to the reactants, which can decrease the induction time [52]; (2) the superb transmission capabilities of the electrons [53]. It is well-known that rGO has a good absorption ability toward reactants and can prevent aggregation of gold NPs. Moreover, high-density dispersion of gold NPs can provide abundant active sites for efficient contact with 4-NP [54]. In brief, combined with the UV–vis absorption spectrum of the rGO/Au catalyst (Figure 6a), when NaBH_4_ was added, the characteristic peak of 4-NP shifted to 400 nm due to the formation of 4-NP ions under alkaline conditions. The change in the peak intensity was negligible in the course of the reaction, which confirmed that the reaction did not proceed without the catalyst. After the addition of the catalysts, the ${BH}_{4}^{-}$ ions and the 4-NP ions were rapidly adsorbed to the active sites of the catalyst. At the same time, the concentration of ${BH}_{4}^{-}$ ions and 4-NP ions on the rGO/Au catalyst increased, which was due to the good absorption ability of rGO. This process would decrease the induction time. In addition, the electrons transferred to the rGO/Au catalyst following the formation of active hydrogen. The active hydrogen was absorbed on the active site of the catalyst, and then 4-NP was attacked and reduced into 4-AP, which was confirmed by the characteristic peak of 4-AP that appeared at 300 nm [54,55]. Finally, 4-AP was desorbed from the catalyst.

## 3. Materials and Methods

### 3.1. Materials

Chloroauric acid tetrahydrate (HAuCl_4_·4H_2_O), chloroplatinic acid (H_2_PtCl_4_·6H_2_O), sodium borohydride (NaBH_4_), hydrazine hydrate, 4-nitrophenol (4-NP), and silver nitrate (AgNO_3_) were obtained from Sinopharm Chemical Reagent Co., Ltd.

### 3.2. Fabrication of rGO-Supported Noble Metal Composites

**Scheme 1:**
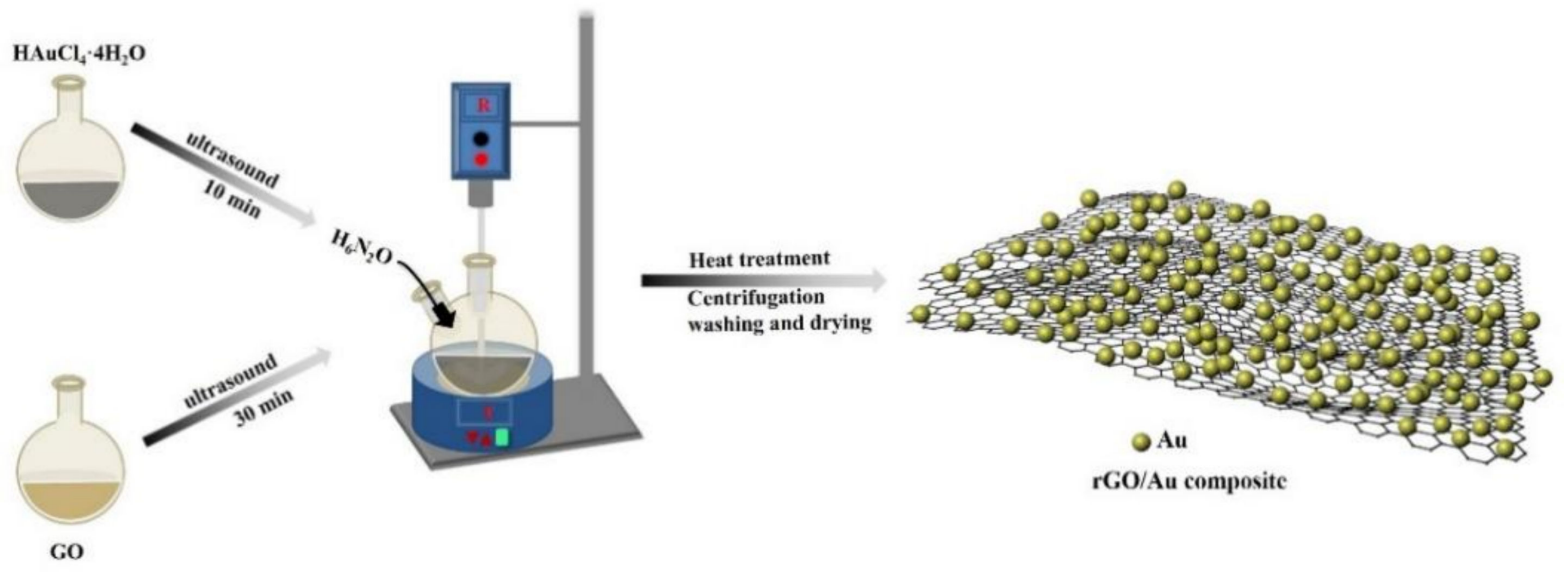
Schematic illustration for the synthesis of the rGO/Au composite.

The composites were fabricated by means of a one-pot facile method using hydrazine hydrate as a reducing agent. In the fabrication of rGO/noble metal nanoparticles, GO (50 mg) and deionized water (50 mL) were ultrasonically dispersed to obtain a GO aqueous solution. Then, HAuCl_4_·4H_2_O (0.5 mmol) and deionized water (10 mL) were ultrasonically dissolved to obtain a chloroauric acid tetrahydrate aqueous solution. The GO aqueous solution was transferred to a two-neck flask, and then the precursor solution of gold was added to GO with mechanical stirring. After 5 min, the above mixture was heated up to 90 °C under mechanical stirring. Finally, the rGO/Au composite was washed and obtained (as shown in Scheme 1). The rGO/Ag and rGO/Pt composites were obtained in the same way.

### 3.3. Applications of rGO-Supported Noble Metal Composites

The rGO-supported noble metal composites were applied for 4-nitrophenol (4-NP) reduction with NaBH_4_. This catalytic reduction process has been widely used to estimate the catalytic properties of a wide variety of functional nanomaterials. The catalytic reduction reaction process was monitored using a UV–visible spectrometer. In this experiment, 4-NP (0.1 mL, 0.005 M), deionized water (2 mL), and NaBH_4_ (1 mL, 0.2 M) were added to a standard quartz cuvette. The characteristic peak of the mixture shifted to 400 nm from 317 nm and the color changed to bright yellow quickly, which confirmed the formation of 4-NP anions. When a catalyst (100 µL, 2 mg/mL) was added, the mixture would become colorless, and the peak intensity would decrease quickly. Moreover, the peak appeared (at 300 nm), which confirmed that 4-AP was formed. The catalytic performances of the catalysts were then compared.

More detailed information on the materials and methods is offered in the Supplementary Materials.

## 4. Conclusions

In summary, noble metal (gold, silver, and platinum) NPs supported on rGO catalysts were successfully prepared in this work through a facile method. The crystalline size of gold NPs was smaller than that of silver NPs and platinum NPs. The *I_D_/I_G_* ratios of rGO/Au, rGO/Ag, and rGO/Pt were calculated to be 1.106, 1.078, and 1.047, respectively. The k value of rGO/Au was higher than those of the rGO/Ag and rGO/Pt, thus demonstrating that the rGO/Au catalyst displayed the best catalytic activity for 4-NP reduction. The higher catalytic activity for rGO/Au may be attributed to the small crystalline size of gold NPs and the unique structural characteristics. This work presents a facile method to synthesize reduced graphene oxide-supported nanostructure catalysts, which may have potential application in catalysis.

## Figures and Tables

**Table 1:** Comparison of the catalytic activity for 4-nitrophenol reduction with different catalysts.

Catalysts	Time (min)	k (min^−1^)	Reference
Au nanorods	32	0.0859	[48]
Cu_2_O@CuO (Au–Pd)	4	1.158	[49]
rGO/Pd_48_Au_52_/PPy	18.5	0.269	[50]
Au nanoparticles/GO	18	0.188	[51]
rGO/Au	8	0.618	This work

## Data Availability

The data presented in this study are available on request from the corresponding author.

## Supplementary Materials

The following are available online. Figure S1: the XRD patterns of GO and rGO, Figure S2: XPS survey spectra of rGO/Au, rGO/Ag, and rGO/Pt.
