# Supplementary material for: One-Pot Facile Synthesis of Noble Metal Nanoparticles Supported on rGO with Enhanced Catalytic Performance for 4-Nitrophenol Reduction
Source: Molecules. 2021 Nov 30;26(23):7261. doi: 10.3390/molecules26237261 (PMC8659260; doi:10.3390/molecules26237261)
Supplement: Supplementary file 1 [file molecules-26-07261-s001.zip › molecules-1471700-supplementary.pdf]

Article

One-Pot Facile Synthesis of Noble Metal Nanoparticles Supported on rGO with Enhanced Catalytic Performance for 4-Nitrophenol Reduction

Xiaolong Zhang [1,2], Shilei Jin [2], Yuhan Zhang [2], Liyuan Wang [2], Yang Liu [2] and Qian Duan [1,*]

[1] School of Materials Science and Engineering, Changchun University of Science and Technology, Changchun 130022, China; zhangxiaolong@jlnu.edu.cn

[2] Key Laboratory of Functional Materials Physics and Chemistry of the Ministry of Education, Jilin Normal University, Changchun 130103, China; jinshilei2001@163.com (S.J.); zhangyuhan193@163.com (Y.Z.); wly18629899813@163.com (L.W.); liuyang@jlnu.edu.cn (Y.L.)

* Correspondence: duanqian88@hotmail.com; Tel.: +86-431-85583015

Abstract: In this study, reduced graphene oxide (rGO)-supported noble metal (gold, silver, and platinum) nanoparticle catalysts were prepared via the one-pot facile co-reduction technique. Various measurement techniques were applied to investigate the structures and properties of the catalysts. The relative intensity ratios of I_D/I_G in rGO/Au, rGO/Ag, rGO/Pt, and GO were 1.106, 1.078, 1.047, and 0.863, respectively. The results showed the formation of rGO and that noble metal nanoparticles were decorated on rGO. Furthermore, the catalytic activities of the designed nanocomposites were investigated via 4-nitrophenol. The catalysts were used in 4-nitrophenol reduction. The catalytic performance of the catalyst was evaluated by the apparent rate constant k values. The k value of rGO/Au was 0.618 min^{-1}, which was higher than those of rGO/Ag (0.55 min^{-1}) and rGO/Pt (0.038 min^{-1}). The result proved that the rGO/Au catalyst exhibited a higher catalytic performance than the rGO/Ag catalyst and rGO/Pt catalyst. The results provide a facile method for the synthesis of rGO- supported nanomaterials in catalysis.

Keywords: 4-nitrophenol; $NaBH_4$; noble metal nanoparticles; catalytic reduction.

1. Materials

Sulfuric acid (H_2SO_4), potassium persulfate ($K_2S_2O_8$), phosphorus pentoxide (P_2O_5), phosphoric acid (H_3PO_4), potassium permanganate ($KMnO_4$), hydrogen peroxide (H_2O_2), and hydrochloric acid (HCL) were obtained from Sinopharm Chemical Reagent Co., Ltd. GO was prepared by a modified Hummers' method from graphite powder [1,2]. All the chemicals were used without further treatment.

References:

1. Hummers, W.S.; Offeman, R.E. Preparation of Graphitic Oxide. *J. Am. Chem. Soc.* **1958**, *80*, 1339.
2. Marcano, D.C.; Kosynkin, D.V.; Berlin, J.M.; Sinitskii, A.; Sun, Z.Z.; Slesarev, A.; Alemany, L.B.; Lu, W.; Tour, J.M. Improved Synthesis of Graphene Oxide. *ACS Nano* **2010**, *8*, 4806–4814.

2. Instrumentation

The morphology and structure were characterized by a TECNAI F20 transmission electron microscope (TEM) at an accelerating voltage of 200 kV with energy dispersive X-ray (EDX) spectroscopy. The crystallite structures of the samples were acquired via a Rigaku D/Max-2500 copper rotating-anode X-ray diffractometer (XRD) using Cu Kα radiation (40 kV, 200 mA, λ = 0.154 nm). Raman spectra were measured using a Renishaw Raman system, model 2000 confocal microscopy spectrometer (London, UK) at a laser wavelength of 514 nm. X-ray photoelectron spectrum was measured with Thermo Scientific ESCALAB 250Xi X-ray photoelectron spectroscopy (XPS). Ultraviolet-visible spectroscopy (UV-Vis) absorbance spectra were recorded on a Shimadzu UVmini-1240 spectrophotometer in the range of 250-500 nm.

Figure S1

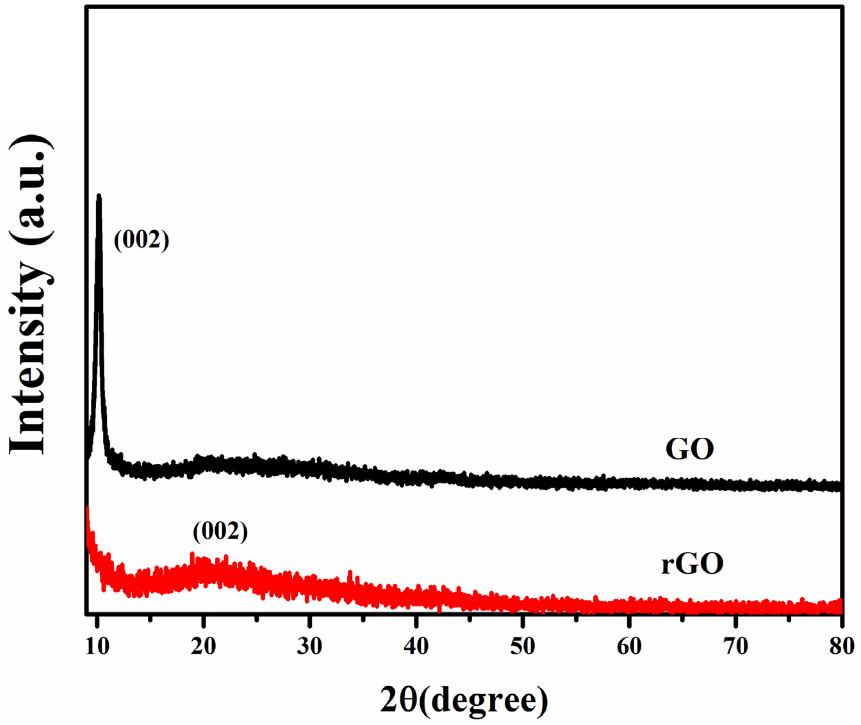

Figure S1. XRD patterns of GO and rGO.

Figure S2

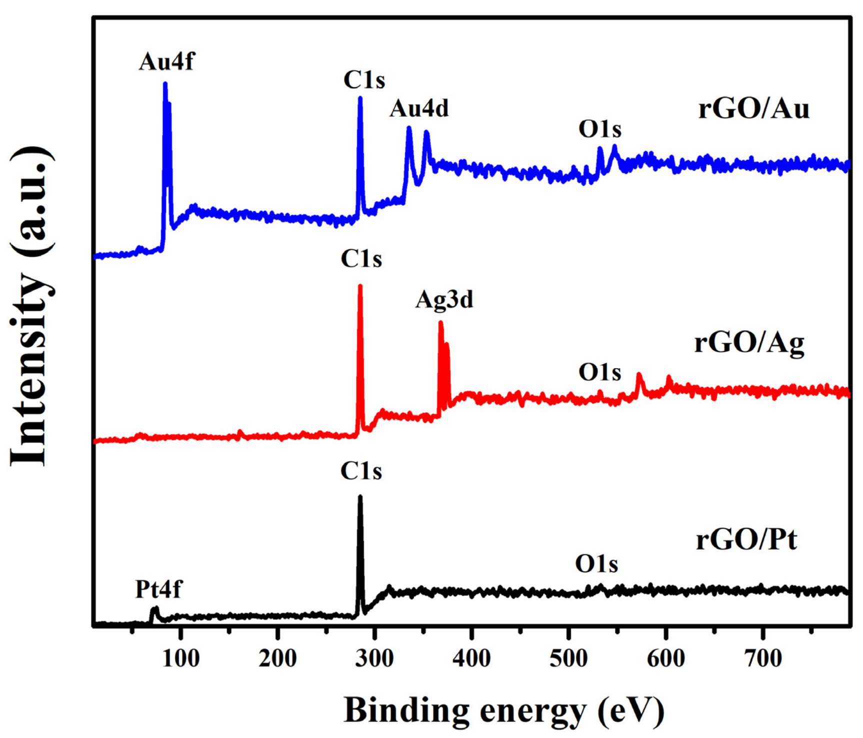

Figure S2. XPS survey spectra of rGO/Au, rGO/Ag, and rGO/Pt.
